# Longer Leukocyte Telomere Length Increases the Risk of Atrial Fibrillation: A Mendelian Randomization Study

**DOI:** 10.14336/AD.2022.02251

**Published:** 2022-10-01

**Authors:** Yaozhong Liu, Yunying Huang, Chan Liu, Qiming Liu

**Affiliations:** ^1^Department of Cardiovascular Medicine, Second Xiangya Hospital, Central South University, Hunan, China.; ^2^Department of International Medicine, Second Xiangya Hospital, Central South University, Hunan, China.

**To the Editor,**

Advanced age is the most critical driving factor for atrial fibrillation (AF) [1]. As a marker for biological aging, telomere shortening is implicated in numerous age-related diseases [2-5]. However, longer telomere length (TL) may also increase the risks of diseases [4, 5]. A few observational studies have investigated the association between TL and the risk of AF [6-9]. These studies provided inconsistent results and were unable to establish causality. Hence, we conducted the two-sample Mendelian randomization (MR) analysis to evaluate the causal role of leukocyte telomere length (LTL) in AF using summary statistics from large-scale genome-wide association studies (GWAS).

The GWAS data for LTL was from UK Biobank (UKB), involving 472,174 participants [5]. LTL was measured as the ratio of telomere repeat copy numbers relative to that of a single-copy gene. The genetic variants in the LTL GWAS have been adjusted for age and sex. Single-nucleotide polymorphisms (SNPs) associated with LTL at genome-wide significance (P<5×10^-8^) were selected. These SNPs were clumped (linkage disequilibrium R^2^=0.01, >10,000kb) to ascertain independence. If a particular SNP is absent in the outcome GWAS, its proxy SNP (R^2^>0.8) is considered as a substitution.

In the primary analysis, the GWAS data of AF was obtained from Nielsen’s study [10], including 60,620 cases and 970,216 controls. Since the UKB individuals were also involved in the AF GWAS, this may have produced weak instrumental bias due to sample overlap. We conducted the secondary analysis, and the latest FinnGen 2021 AF GWAS (22,068 cases and 116,926 controls) was used as the outcome data. The MR analyses were performed by Two-Sample MR and Mendelian Randomization packages [11, 12]. The study design was approved by the Institutional Committee on Human Research at the Second Xiangya Hospital. Ethical approval was obtained for all original studies.

**Figure 1.**
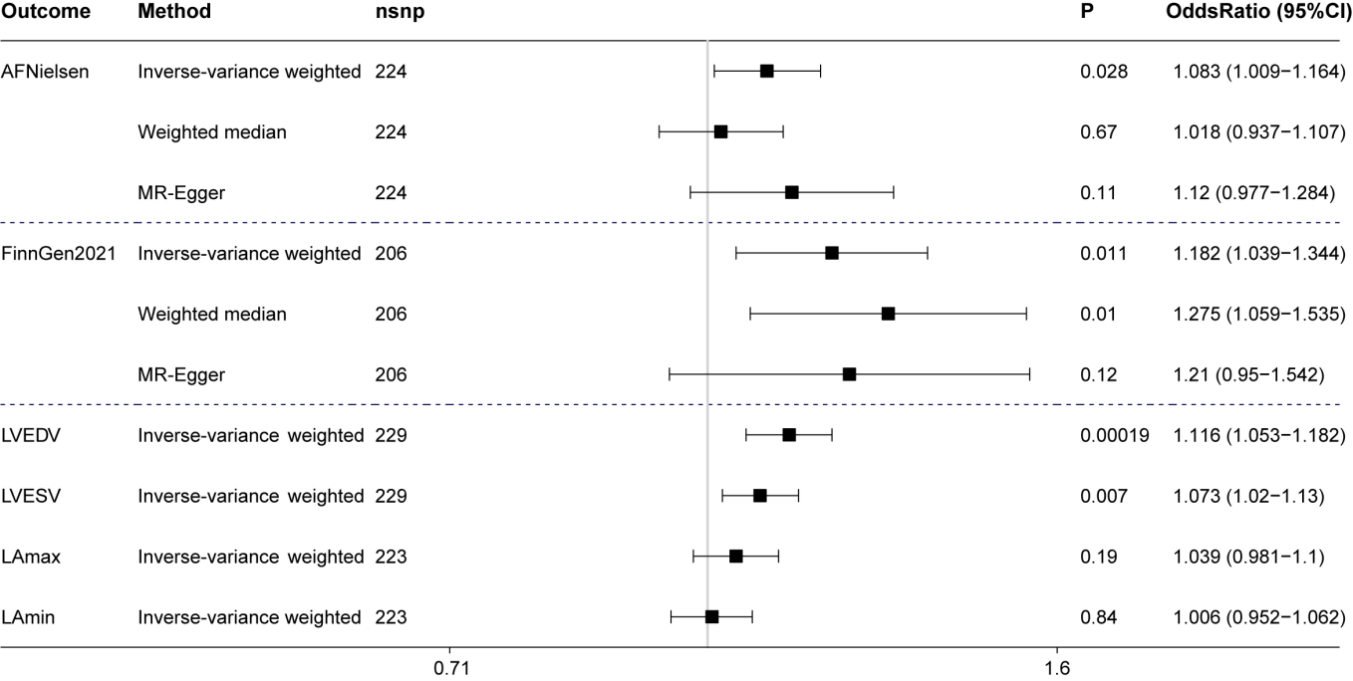
**AF and heart volume associated with genetically determined LTL**. Odds ratios are expressed per 1-SD increase in genetically determined LTL. AF, atrial fibrillation; LTL, leukocyte telomere length; LVEDV, indexed left ventricular end-diastolic volume; LVESV, indexed left ventricular end-systolic volume; LAmax, indexed left atrial maximum volume; LAmin, index left atrial minimum volume; CI, confidence interval.

In the main MR analysis (inverse-variance weighted method), longer genetically determined LTL increased risk of AF in Nielsen’s study (odds ratio [OR], 1.083 [95% CI, 1.009-1.164]; P=0.028) and in FinnGen consortium (OR, 1.182 [95% CI, 1.039-1.344]; P=0.011). Sensitivity analyses (MR-Egger and weighted median method) provided less precise estimates but had consistent directions (Fig. 1). There is no directional pleiotropy (P-Egger intercept > 0.05) or reverse causality.

Heart enlargement has been implicated in the development of AF. The Framingham heart study [13] showed that LTL was positively associated with left ventricular mass. The causal relationship between LTL and heart volume was then investigated using the following GWAS summary data: indexed left ventricular end-diastolic volume (LVEDV, n = 36,041), indexed left ventricular end-systolic volume (LVESV, n=36,041) [14], indexed left atrial maximum volume (LAmax, n= 35,658), and index left atrial minimum volume (LAmin, n=35,658) [15]. Our results confirmed that longer LTL had effect on increasing LVEDV (OR, 1.116 [95% CI, 1.053-1.182], P = 0.00019) and LVESV (OR, 1.073 [95% CI, 1.02-1.13], P = 0.007). No significant causality was detected between LTL and left atrial volume.

Longer telomere length is generally considered a protective factor for cardiovascular diseases [3, 4]. However, we found that longer LTL increases the risk of AF. This finding could partly be attributed to the effect of longer LTL on increasing left ventricular volume[13]. Longer LTL probably promoted the proliferation of residual progenitor cells and then contributed to hypertrophy of the left ventricle. Likewise, this phenomenon was supposed to happen in the left atrium, despite the association between LTL and left atrial volume is not significant. Besides, a previous MR study [5] showed that longer LTL increased blood pressure, insulin-like growth factor-1 level, waist-hip ratio, and triglycerides level. These metabolic risk traits may mediate the effect of longer LTL on incident AF. Interestingly, our results could also explain why athletes are more vulnerable to AF since high-density physical activity could increase LTL. Further validation is required.

This study has some limitations. First, the results of the MR study could be disturbed by pleiotropy when genetic instruments affect the outcome through other exposure. However, no directional pleiotropy was detected, and sensitivity analyses yielded convincing results. Second, all participants of the GWASs were of European ancestry. Our finding may not apply to other races. Third, we can’t investigate the non-linear relationship between LTL and AF because of lacking individual data of LTL.

Our MR study found that longer LTL may increase the risk of AF, suggesting that longer LTL should be regarded as a risk factor of AF instead of a protective one. Further studies are still required to confirm our findings. It would be necessary to determine whether the LTL is associated with the severity of AF.
